# Recombinant activated factor VII (rFVIIa) in refractory haemorrhage for non-haemophiliacs: an eleven-year single-centre experience

**DOI:** 10.1186/s12878-018-0126-z

**Published:** 2018-11-23

**Authors:** Nurfatin Mohd Shah, Soon Eu Chong, Syahirah Mohamed Yusoff, Mohd Zulfakar Mazlan, Khairul Bariah Johan, Nizuwan Azman, Jo Anne Lim, Siti Mardhiana Mohamad, Siti Salmah Noordin, Zainab Abdul Ghaffar, Mohd Hasyizan Hassan, Muhammad Azrul Zabidi, Nur Arzuar Abdul Rahim

**Affiliations:** 1 Regenerative Medicine Cluster, Advanced Medical and Dental Institute, Universiti Sains Malaysia, 13200 Kepala Batas, PNG Malaysia; 2 School of Medical Sciences, Universiti Sains Malaysia, 16150 Kubang Kerian, KTN Malaysia; 3 Hospital Universiti Sains Malaysia, 16150 Kubang Kerian, KTN Malaysia; 4 Department of Internal Medicine, Hospital Sultan Abdul Halim, 08000 Sungai Petani, KDH Malaysia; 5 Department of Pharmacy, Hospital Universiti Sains Malaysia, 16150 Kubang Kerian, KTN Malaysia; 6 Integrative Medicine Cluster, Advanced Medical and Dental Institute, Universiti Sains Malaysia, 13200 Kepala Batas, PNG Malaysia

**Keywords:** Recombinant activated factor VII, rFVIIa; massive bleeding, Off-label, Non-haemophilia

## Abstract

**Background:**

Massive bleeding is one of the commonest salvageable causes of death. The search for an ideal haemostatic agent during massive bleeding is still ongoing. One of the novel haemostatic medications is recombinant activated factor VII (rFVIIa). To date, the usage of rFVIIa during massive haemorrhage among non-haemophiliac patients remains off-label. The aim of this study is to report our experience in using rFVIIa to treat refractory bleeding.

**Methods:**

Medical records of all patients treated with rFVIIa for massive bleeding over an eleven-year period in a single institution were recorded. Treatment indications, 24-h and 30-day mortality, changes in transfusion needs and coagulation profiles after rFVIIa administration were analysed.

**Results:**

rFVIIa were administered in 76 patients. Of these, 41 (53.9%) were non-surgical bleeding, followed by 22 patients (28.9%) with trauma, other surgery bleedings in 9 patients (11.8%) and 4 patients (5.4%) with peripartum haemorrhage. Total survival rate was 78.9% within 24 h and 44.7% over 30 days. Among all these patients who had received rFVIIa due to life-threatening haemorrhage, blood and blood product requirements were significantly reduced (*P* < 0.001), and the coagulation profiles improved significantly (*P* < 0.05). Two patients with preexisting thromboembolism were given rFVIIa due to intractable bleeding, both survived. No thromboembolic events were reported after the administration of rFVIIa.

**Conclusions:**

rFVIIa significantly improved coagulation parameters and reduced blood product requirements during refractory haemorrhage. Additionally, usage of rFVIIa in trauma and peripartum haemorrhage patients yield better outcomes than other groups of patients. However, the overall mortality rate remained high.

## Background

Massive bleeding is one of the commonest salvageable causes of death [1] thus the searches for an ideal haemostatic agent during massive bleeding persist [2]. One of the novel haemostatic medications is recombinant activated factor VII (rFVIIa; Novo Nordisk, Bagsvaerd, Denmark). It was originally developed for haemophilia A and B patients with inhibitors to factor VIII or IX by bypassing the intrinsic pathway of coagulation cascade [3].

rFVIIa acts via two mechanisms. First, it acts directly with tissue factor released at the sites of vascular disruption and activates the common coagulation cascade via activated factor X (Xa). rFVIIa also binds to activated platelets, which concentrates factor X activation to sites of tissue injury. The factor Xa generated by these two mechanisms ultimately drives the thrombin burst which cleaves fibrinogen to fibrin thereby initiating the formation of a fibrin meshwork and clot stabilization [4].

Presently, evidence available for rFVIIa in non-haemophiliacs is not consistent [5–7] with a major concern being its potent thromboembolic property [7]. Thus, the usage of rFVIIa among non-haemophiliac patients remains off-label [8]. However, in cases of intractable bleeding, rFVIIa may still be a last resort of life-saving treatment [9–12]. In fact, rFVIIa has been declared as a standard of care by the United States Army in battlefield medicine to treat severe bleeding due to war-wound [13, 14].

Currently, most data available were contributed by developed countries. The authors aimed to report their eleven-year outcome on off-label rFVIIa usage in treating refractory bleeding in a fast-developing country.

## Methods

This retrospective study was conducted at Hospital Universiti Sains Malaysia, Malaysia. Study samples were non-haemophiliac patients who had received rFVIIa treatment for massive bleeding from 1st January 2006 to 31th December 2016. Medical records of patients were traced from pharmacy and blood bank database, reviewed, and grouped into trauma, peripartum, surgery bleedings and medical causes of bleeding. Inclusion criteria were patients with loss of one blood volume within 24 h, loss of 50% blood volume within three hours, or patients who bled at the rate exceeding 150 ml/min, who have received rFVIIa treatment. Haemophilia patients and subjects with incomplete record documentation were excluded. Data collected include underlying conditions and treatment, indication for blood transfusion, rFVIIa dosage, blood pressure readings, haemoglobin levels, arterial pH, temperatures, coagulation profiles, and blood product requirements before and after administration of rFVIIa. Thromboembolic complications, duration of stay, survival rates at 24-h and day-30 after administration of rFVIIa were also recorded.

### Statistics

Quantitative data were expressed as mean ± standard deviation (SD) and median (interquartile range, IQR). For comparison before and after of administration of rFVIIa, results were performed using paired *t*-test for normally distributed data. For skewed data, Wilcoxon Signed-Rank test was used for analysis. Differences were considered significant at a *p* < 0.05. All statistical calculations were done using Microsoft Excel and Statistical Package for the Social Science (SPSS) software Version 22. For the outcome of survival and thromboembolic complications, data were analysed descriptively.

## Results

During the eleven-year study period, 76 non-haemophiliac patients received rFVIIa for massive haemorrhage. The demographic data, major bleeding categories and rFVIIa dose were shown in Table 1. 78.9% of the patients survived at 24-h after rFVIIa administration while only 44.7% survived for at least 30-days. (Table 2a).

**Table 1:** Patients characteristics and doses

Indications	n	Age (Mean ± SD)	Weight (kg) (Mean ± SD)	Gender (%)	Dose (mcg/kg) (Mean ± SD)
All occasions	76	37.93 ± 18.25	61.21 ± 12.28	Male (61.8) Female (38.2)	83.38 ± 24.80
Trauma	22	30.00 ± 15.60	61.57 ± 12.85	Male (81.8) Female (18.2)	92.39 ± 31.68
Peripartum	4	35.75 ± 5.62	59.13 ± 15.70	Male (0) Female (100)	92.08 ± 5.30
Surgery bleedings	9	43.00 ± 15.58	51.64 ± 11.86	Male (71.4) Female (28.6)	77.27 ± 24.21
Medical	41	41.07 ± 19.91	63.42 ± 11.28	Male (56.1) Female (43.9)	79.04 ± 20.66

*SD* Standard deviation

**Table 2:** Breakdown of patients in detail

(a) for all occasions							
	24-hour outcome		Day-2 – Day-30 outcome			Overall 30-day outcome	
Total n	Survivors n (%)	Deaths n (%)	Survivors n (%)	Deaths n (%)		Survivors n (%)	Deaths n (%)
76	60 (78.9)	16 (21.1)	34 (56.7)	26 (43.3)		34 (44.7)	42 (55.3)
(b) according to causes of bleeding							
Indications	Total	24-hour outcome		Day-2 – Day-30 outcome		Overall 30-day outcome	
	n	Survivors n (%)	Deaths n (%)	Survivors n (%)	Deaths n (%)	Survivors n (%)	Deaths n (%)
Trauma	22	17 (77.3)	5 (22.7)	11 (64.7)	6 (35.3)	11 (50)	11 (50)
Peripartum	4	3 (75)	1 (25)	3 (100)	0 (0)	3 (75)	1 (25)
Surgery bleedings	9	7 (77.8)	2 (22.2)	3 (42.9)	4 (57.1)	3 (33.3)	6 (66.7)
Medical	41	33 (80.5)	8 (19.5)	17 (51.5)	16 (48.5)	17 (41.5)	24 (58.5)

rFVIIa was administered as intravenous bolus at doses varying between 30 mcg/kg to 112 mcg/kg. The doses were determined by clinicians, either haematologists, anaesthesiologists or intensive care physicians. A single rFVIIa dose was used in 72 patients (94.7%). Four patients received two doses which were given three hours apart. The mean rFVIIa dose administered was 83.38 ± 24.80 mcg/kg. Majority of the patients given rFVIIa were due to medical causes of bleeding (53.9%) which involved complications from leukemia, lymphoma, liver failure, multiple myeloma, severe dengue, leptospirosis, pulmonary haemorrhage, sepsis with disseminated intravascular coagulopathy and even iatrogenic causes of bleeding. This was followed by trauma (28.9%), surgery bleedings (11.8%) and peripartum haemorrhage (5.4%) (Table 1).

The last readings of blood pressure, temperature, and haemoglobin recorded before administration of rFVIIa were taken as “pre-rFVIIa” readings. The same parametres recorded 24 h after administration of rFVIIa were taken as “post-rFVIIa” readings. There were slight increases in mean arterial pressure, arterial pH and temperature 24 h after the rFVIIa administration, however the increments were not statistically or clinically significant.

Prior to rFVIIa administration, patients had received 3.76 ± 4.64 units of packed red blood cells, 4.20 ± 3.17 units of fresh frozen plasma, 3.50 ± 3.66 units of platelet concentrate and 3.47 ± 4.44 units of cryoprecipitate. The requirements for packed cells and blood products after administration of rFVIIa were significantly reduced (*P* < 0.001) among the patients who had received rFVIIa. Overall, there were no significant changes of haemoglobin level before and after rFVIIa administration, except in the group of trauma, in which the haemoglobin level increased significantly (Table 3).

**Table 3:** Blood transfusion requirement and haemoglobin before and after initial dose of rFVIIa

	PRBC Median (IQR)			FFP Median (IQR)			PLT Median (IQR)			CRYO Median (IQR)			HB Median (IQR)		
	Before	After	*p*-value	Before	After	*p*-value	Before	After	*p*-value	Before	After	*p*-value	Before	After	*p*-value
All occasions (*n* = 76)	3.76 ± 4.64^#^	1.16 ± 1.81^#^	< 0.001^#^	4.20 ± 3.17^#^	1.14 ± 1.73^#^	< 0.001^#^	3.50 ± 3.66^#^	1.66 ± 1.71^#^	< 0.001^#^	3.47 ± 4.44^#^	1.11 ± 2.38^#^	< 0.001^#^	8.08 ± 2.29^#^	8.68 ± 3.53^#^	0.166^#^
Trauma (*n* = 22)	2.00 (4.50)	1.00 (2.00)	0.005^*^	4.00 (5.50)	0.00 (4.00)	0.001^*^	1.50 (6.50)	1.33 (4.00)	0.091^*^	4.00 (6.00)	0.00 (4.00)	0.024^*^	8.25 (3.05)	9.60 (1.63)	0.005^*^
Peripartum (*n* = 4)	0.00 (1.50)	1.00 (2.00)	0.317^*^	4.00 (3.00)	0.00 (0.00)	0.083^*^	2.50 (3.75)	1.00 (2.00)	0.357^*^	0.00 (4.50)	0.00 (0.00)	0.317^*^	9.55 (5.00)	10.65 (2.40)	0.066^*^
Surgery bleedings (*n* = 9)	2.00 (7.00)	1.00 (0.50)	0.075^*^	4.00 (4.00)	0.00 (4.00)	0.054^*^	3.00 (3.50)	2.00 (3.50)	0.798^*^	2.00 (8.00)	0.00 (1.06)	0.043^*^	7.50 (5.30)	11.90 (4.20)	0.138^*^
Medical (*n* = 41)	3.95 ± 4.70^#^	1.05 ± 1.09^#^	< 0.001^#^	4.17 ± 2.55^#^	1.10 ± 1.71^#^	< 0.001^#^	3.66 ± 3.55^#^	1.50 ± 1.72^#^	< 0.001^#^	3.17 ± 4.28^#^	0.95 ± 2.24^#^	0.005^#^	7.94 ± 2.23^#^	7.76 ± 3.77^#^	0.776^#^

^#^Values expressed as mean ± SD (standard deviation), using Paired sample *t*-test

*Values expressed as median (IQR) using Wilcoxon Signed Rank test

*PRBC* Packed red blood cells; *FFP* Fresh frozen plasma; *PLT* Platelet; *CRYO* Cryoprecipitate; *HB* Haemoglobin; *rFVIIa* Recombinant activated factor VII

There were significant differences in all coagulation profiles before and after administration of rFVIIa. The Prothrombin time (PT) of patients improved from 24.02 ± 21.79 s to 12.50 ± 5.20s (*p* < 0.001). There were also significant improvements in activated partial thrombin time (aPTT) from 52.31 ± 25.13 s to 39.48 ± 18.32 s (*p* = 0.001) (Table 4).

**Table 4:** Coagulation parameters before and after initial dose of rFVIIa

	INR (Mean ± SD)			PT (seconds) (Mean ± SD)			aPTT (seconds) (Mean ± SD)		
	Before	After	*p*-value	Before	After	*p*-value	Before	After	*p*-value
All patients (*n* = 76)	1.55 ± 0.42	0.97 ± 0.44	< 0.001	24.02 ± 21.79	12.50 ± 5.20	< 0.001	52.31 ± 25.13	39.48 ± 18.32	0.001
Surgical (*n* = 35)	1.64 ± 0.44	0.84 ± 0.47	< 0.001	24.81 ± 24.45	11.02 ± 5.49	0.003	57.84 ± 33.78	37.42 ± 20.23	0.009
Medical causes (*n* = 41)	1.47 ± 0.39	1.09 ± 0.39	< 0.001	23.35 ± 19.53	13.77 ± 4.63	0.006	47.60 ± 12.84	41.24 ± 16.56	0.021

*SD* Standard deviation

*INR* International normalized ratio; *PT* Prothrombin time; *aPTT* Activated partial thromboplastin time; *rFVIIa* Recombinant activated factor VII

More than three-quarter or sixty (78.9%) of the massive bleeding patients survived at 24-h after the administration of rFVIIa. Among these 60 survivors, 34 of them (56.7%) survived after 30 days (Table 2a). Patients who suffered massive bleeding due to peripartum haemorrhage and trauma yield the best 30-day survival rate at 25% and 50% respectively. Among the nine non-trauma surgery patients who received rFVIIa due to refractory haemorrhage (coronary artery bypass grafting, craniectomy for tumour debulking surgeries, thoracotomy, upper gastrointestinal bleeding surgery and tumour excision), two (22.2%) passed away within 24 h and only three patients (33.3%) survived more than 30 days (Table 2b).

There was no thromboembolic event recorded after rFVIIa administration in the institution. The occurrence of thromboembolic event was tracked from medical record based on clinicians’ diagnosis. Evaluation and investigations involved include renal function test, liver function test, D-Dimer for suspected patients, electrocardiography (ECG), doppler ultrasound, echocardiography, computed tomography pulmonary angiography (CTPA), which was done upon request by clinicians. There were two patients who had developed pulmonary embolism prior to the intractable bleeding episode, i.e. before the administration of rFVIIa. Surprisingly, both survived without any complications.

## Discussion

Massive bleeding is one of the reversible causes of morbidity and mortality. Rapid control of bleeding may prevent further complications and subsequently yield a better outcome [15]. This study shows that the administration of rFVIIa had significantly reduced the blood product requirements and improved the coagulation parametres in all the patients who had received rFVIIa, including the survivors and among patients who had succumb to death. This result is comparable to studies reported by Palmason et al. [9] and Soliman et al. [16]. Minimizing blood components transfusion is important as massive transfusion may lead to dilutional coagulopathy [17], disseminated intravascular coagulopathy [18], extreme fibrinolysis, hypothermia and acidosis [19] which may worsen the situation. By correcting both the coagulation profiles and blood product requirements, haemostasis can be achieved more effectively.

Overall, the haemoglobin (Hb) levels increased after rFVIIa infusion, except in the medical patients where the Hb dropped (Table 3). A possible explanation is the differences of approach in transfusion for surgical bleeding and medical causes of bleeding. For bleeding due to surgical causes, securing bleeding and transfusion is the mainstay of treatment, and occasionally transfusion will still be on-going after bleeding was successfully secured.

On the other hand, the medical patients had a lower starting haemoglobin because of their underlying diseases e.g. haematological malignancy, lymphoma or anaemia of chronic diseases. It was generally accepted that this group of patients could tolerate a lower Hb level. Thus, for bleeding due to medical causes, observation is one of the major components in the management. It was also hypothesised that physicians allow a higher threshold of transfusion in many of the occasions.

Patients recruited in this study were in dire states of massive bleeding due to the institution’s policy of administering rFVIIa only as a last resort. However, their 24-h and 30-day mortality rates of severe intractable bleeding were still comparable to the general massive haemorrhage (which might be not as severe as an “intractable bleeding”) population in the developing countries [20, 21]. There was no clear protocol to guide the doses of rFVIIa in this institution, in which the doses were mainly depend on clinician preference according to available references and individualised justifications.

There were more patients died later at day-30 compared to 24-h post rFVIIa infusion (Table 2a). This was because even though bleeding was overcome, the patients were suffering from severe morbidity. Most of the patients (54.8%) who passed away from 24-h to 30-day post rFVIIa were due to unresolved sepsis with multiorgan failure or hospital acquired infections. Another three of them were advanced blood malignancy patients whom family had opted for do not resuscitate (DNR) order after the bleeding occasion. Meta-analysis has shown that rates of hospital acquired infection at the intensive care units in developing countries were at least three times higher than from the developed countries [22]. This was especially true among the medical patients in this study who were post-chemotherapy, immunosuppressed and prone to infection. Other patients who succumbed to death include one patient with cardiogenic shock, one with severe dengue, two severe liver injuries, three patients with severe brain injuries and another two were due to recurrent intracranial bleed.

On the other hand, observation from this study also shows that patients who bled due to peripartum haemorrhage and trauma yield a better 30-day survival rate. Unlike medical patients who did not suffer any obvious vessel damage, rFVIIa may be a suitable intervention for bleeding in trauma and peripartum patients due to its localised effect at the site of injury [23].

A drawback of rFVIIa is the risk of thromboembolism [6, 24]. However, there were no thromboembolic complications reported in this study. This was probably due to the relatively small number of rFVIIa usage in a single centre. Besides, majority of the patients were only given a single dose of rFVIIa, and most of the doses were not exceeding the current consensus of 90 mcg/kg for refractory bleeding among the non-haemophiliac population [12]. The patients who were given more than one dose of rFVIIa were relatively young. They were associated with a lower risk of thromboembolic event after rFVIIa administration, as reported by previous literature [6]. Nevertheless, low detection rate and less facility for the diagnosis of thromboembolic event could also be a cause.

Interestingly, there were two patients who had suffered thromboembolism prior to the administration of rFVIIa. Both patients survived without any thromboembolic complications. The first patient suffered from pulmonary embolism six weeks prior to her hysterectomy for placenta percreta. She developed intractable bleeding after an emergency uterine artery embolization prior to the hysterectomy. The second patient had a thyroid carcinoma with submassive pulmonary embolism before he suffered an iatrogenic massive haemothorax during pleural tapping. This is again explained by the localised effect of rFVIIa at the site of injury [25]. Studies have shown that rFVIIa induce haemostasis at the vessel injury site by complex formation, without activation of systemic coagulation pathway [19, 26, 27].

There are some limitations that need to be addressed in this study. Firstly, there was no control group. The sample size was small due to the limited number of available patients. For the same reason, the type of surgery conducted was limited. Certain types of patients who are usually given off-label rFVIIa were not being studied, such as liver transplantation and overwarfarinization. The reliability of the study is also limited by the retrospective nature of data collection. However, it is difficult and almost impossible to conduct a prospective randomised-control trial among these critically bleeding patients. Further multicenter case-control collaboration could provide more evidence on the outcome of off-label rFVIIa usage.

Additionally, the policy of the institution to administer rFVIIa only as a last resort may have also contributed to its drawback. The timing of rFVIIa administration is related to its efficacy. “Last-ditch” use of rFVIIa in severe bleeding is believed to be ineffective [28]. As rFVIIa acts locally at vascular injury sites [25], it may have an earlier role in haemostatic stability in the acute bleeding patients.

## Conclusion

This report reported some benefits of intervention with rFVIIa i.e. a reduction in the amount of transfusion requirement and improvement of coagulation profiles. As optimal timing of rFVIIa administration is yet to be established, further studies should be conducted to assess the benefit of rFVIIa given at an earlier stage rather than as a last resort when a patient had gone into the uncompensated phase of haemorrhage.

## Abbreviations

aPTT: Activated partial thrombin time
CRYO: Cryoprecipitate
HB: Haemoglobin
IQR: Interquartile range
PLT: Platelet
PT: Prothrombin time
rFVIIa: Recombinant activated factor VII
SD: Standard deviation
SPSS: Statistical Package for the Social Science
Xa: Activated factor X
